# Splanchnic Vein Thrombosis in the Mediterranean Area in Children

**DOI:** 10.4084/MJHID.2011.027

**Published:** 2011-07-08

**Authors:** Hanaa El-Karaksy, Mona El-Raziky

**Affiliations:** The Department of Pediatrics, Cairo University, Egypt

## Abstract

Abdominal venous thrombosis may present as splanchnic venous thrombosis (SVT) (occlusion of portal, splenic, superior or inferior mesenteric veins) or Budd- Chiari Syndrome (BCS) (thrombosis of inferior vena cava and/or hepatic veins). The aim of this review is to report the scanty data available for SVT in the South Mediterranean area. In one Egyptian study, the possible circumstantial risk factors for portal vein thrombosis (PVT) were found in 30% of cases: 19% neonatal sepsis, 8.7% umbilical catheterization, 6% severe gastroenteritis and dehydration. Another Egyptian study concluded that hereditary thrombophilia was common in children with PVT (62.5%), the commonest being factor V Leiden mutation (FVL) (30%). Concurrence of more than one hereditary thrombophilia was not uncommon (12.5%). The first international publication on hepatic veno-occlusive disease (VOD) in Egypt was in 1965 in children who rapidly develop abdominal distention with ascites and hepatomegaly. This disease was more frequent in malnourished children coming from rural areas; infusions given at home may contain noxious substances that were hepatotoxic and infections might play a role. VOD of childhood is rarely seen nowadays. Data from South Mediterranean area are deficient and this may be attributable to reporting in local medical journals that are difficult to access. Medical societies concerned with this topic could help distribute this information.

## Introduction:

In general practice, physicians pay extra attention to the thrombosis of coronary, pulmonary, mesenteric or cerebral circulation but not to abdominal venous circulation. Thrombotic occlusion of all major vessels of abdominal cavity has severe clinical consequences and chronic complications.^1^

Thrombosis in the major vessels of the abdomen causes a wide spectrum of clinical pictures ranging from a totally asymptomatic patient to a patient with acute abdominal pain and even impending liver failure in patients with underlying chronic liver disease.^1^ Thrombosis involving the liver vasculature is rare but constitutes a potentially life-threatening situation.^2^

Abdominal venous thrombosis may present as Budd- Chiari Syndrome (BCS) (thrombosis of inferior vena cava and/or hepatic veins) or splanchnic venous thrombosis (SVT) (occlusion of portal, splenic, superior or inferior mesenteric veins). Hereditary and acquired risk factors have been implicated in the etiopathogenesis of abdominal venous thrombosis.^3^,^4^

Normal coagulation hemostasis involves the interaction of an initial vascular reaction (vasoconstriction), thrombocytic activation (white clot formation) and formation of thrombin via activation of coagulation cascade. The balance between the forces favoring formation of a clot and forces against it is the normal state which is controlled with very delicate systems.^1^

Predisposition to venous and occasionally arterial thromboembolism is termed thrombophilia.^5^,^6^,^7^,^8^

Thrombophilia is a term which is proposed as an opposite term against hemophilia. Thrombophilia can be defined as a disturbed state of the coagulation-fibrinolysis balance in favor of thrombosis formation (congenital or acquired in adult life) in which thrombosis (in arterial and/ or venous vasculature) is observed more frequently than normal population. For the gastroenterologists and surgeons, the congenital and acquired causes of the thrombophilia are important not only due to their potential risks to patients’ lives but also their preventability with the advent of genetic tests and surveillance and their treatability with new invasive techniques and new anticoagulant drugs. Thrombophilia can be grouped under two major headlines: inherited and acquired.^1^

Inherited thrombophilias include: factor V Leiden mutation, prothrombin gene mutation, protein C and protein S deficiencies, antithrombin deficiency. While acquired causes of thrombophilia include: oral contraceptive use, pregnancy, puerperium, surgery, immobilization and anti-phospholipid syndrome. Age is considered as an independent risk factor for venous thrombosis mainly due to inactivity, co-morbid illnesses and degenerative changes. Malignant disease is also important; with thrombosis related deaths ranking in the second place among all causes of death in this patient population.^9^,^10^,^11^ Myeloproliferative neoplasms (MPN) form a group of special blood disorders which may be termed as half-malignant due to their natural course and these patients frequently suffer from venous thrombosis. Thrombocytosis and the increased hematocrit, which are natural characteristics of these disorders, also cause thrombosis in the venous systems.^12^ A substantial number of patients with SVT, especially portal vein thrombosis (PVT) were found to be carriers of mutation in Janus kinase 2 (*JAK2*) in the absence of overt signs of chronic myeloproliferative disease.^13^

Both abdominal venous thrombosis, BCS (thrombosis of inferior vena cava and/or hepatic veins) and SVT (occlusion of portal, splenic, superior or inferior mesenteric veins) have been reported in children. However, reports in children are scanty and few studies coming from the South Mediterranean region have comprehensively evaluated prothrombotic risk factors in BCS and PVT in children. The aim of this review is to report the scanty data available for SVT in the South Mediterranean area.

## Portal Vein Thrombosis (PVT):

The portal vein is considered the backbone of the portal venous system that allows for blood from the digestive organs to flow towards the liver. PVT refers to the total or near total obstruction of blood flow secondary to thrombus formation. This thrombus may extend towards liver involving intrahepatic portal veins or may extend distally to involve splenic veins or mesenteric veins. On some occasions extensive involvement of all of these vessels may occur with an increased risk of intestinal ischemia. Therefore, the involved segment of portal venous system and the degree of compensatory mechanisms determine the clinical presentation. PVT has considerable consequences for the liver. Upon cessation of blood flow liver loses about two thirds of its blood supply. Interestingly, while the acute arterial blockage usually results in severe hepatic failure or death, PVT is tolerated well and the patients are almost asymptomatic due to compensatory mechanisms. First compensatory mechanism is the well known “arterial vasodilation” of the hepatic artery (which is a vascular reflex seen in every dual-vessel supplied organ) also observed during portal vein clamping in liver surgery.^14^ This “arterial rescue” stabilizes the liver functions at a normal level in the very acute stages of PVT. The second rescue mechanism is the “venous rescue” involving rapid development of collateral vessels to bypass the obstructed segment. These vessels begin to form in a few days after the obstruction and organize into a cavernomatous transformation in 3–5 wk.^15^,^16^ Acute PVT can rather be called “acute mesenteric thrombosis” due to extensive co-involvement of the superior mesenteric vein and branches. This condition has very acute deleterious effects over intestinal circulation compromising the patient’s life before development of portal hypertension and its consequences.^1^ Besides these compensatory mechanisms, liver bears the burden of decreased blood to some extent. The decreased portal blood flow stimulates apoptosis in hepatocytes of rats when portal vein is obliterated in a gradual fashion^17^ and increases mitotic activity of hepatocytes in the unaffected lobe. The latter effect is well known from selective pre-surgery portal vein obliteration performed in an intention to stimulate the hypertrophy of the opposite lobe of the liver used in cancer surgery. Gradual loss of hepatic mass may be responsible for the occurrence of mild to moderate degree of hepatic synthetic dysfunction observed in advanced stages.

The term PVT refers to thrombosis developing in the trunk of the portal vein from where it can extend toward the liver affecting the right and the left branches. It can also extend to the splenic and the superior mesenteric veins.^18^ Thrombosis of the portal venous system refers to thrombosis at any of the above sites.^19^,^20^ Extrahepatic portal vein obstruction (EHPVO) with cavernous transformation is an important cause of portal hypertension in children. It is associated with gastrointestinal bleeding mainly from oesophageal and/or gastric varices.^21^ In Asia, EHPVO is a common cause of portal hypertension, accounting for 30% of all variceal bleeding, and is the leading cause of variceal bleeding in children.^22^

## Etiology:

The precise etiology of the development of EHPVO in the majority of these children is unknown. The predisposing factors are believed to be the following: conditions that directly injure the vessel, rare portal vein congenital anomalies, and a group of systemic causes such as neonatal sepsis, abdominal sepsis, dehydration, multiple exchange transfusions, and hypercoagulable states. Prothrombotic disorders include deficiencies in the naturally occurring anticoagulants (protein C, protein S, and antithrombin) and factor V Leiden and prothrombin gene mutations (PT20210 G/A). Acquired prothrombotic disorders include the antiphospholipid syndrome, paroxysmal nocturnal haemoglobinuria, and MPNs: polycythaemia vera (PV), essential thrombocythaemia (ET), and idiopathic myelofibrosis.^23^ Recent reports confirm that the JAK2 V617F mutation is strongly implicated in the pathogenesis of myeloproliferative disorder that may be associated with increased rates of thrombosis.^24^,^25^

The possible circumstantial risk factors for EHPVO were analyzed in 69 children presenting to our Pediatric Hepatology Unit at Cairo University, between 1995 and 1997 and we found that 21 (30%) had past history relevant to the etiology of EHPVO: 19% had history of neonatal sepsis, 6 (8.7%) underwent umbilical catheterization for exchange transfusion, 4 (6%) had at least one episode of severe gastroenteritis and dehydration. None had a history of any abdominal surgery or trauma.^26^

The risk of development of PVT was studied at the neonatal intensive care (NICU) in our hospital in newborns that underwent umbilical vein catheterization for exchange transfusion. The study included 128 newborns presenting to NICU, from January 1996–December 1996, with severe neonatal indirect hyperbilirubinemia with or without septicemia. Two out of 128 developed PVT as seen in the first Doppler study during their stay in NICU. Portal vein cavernoma, denoting collaterals around the occluded portal vein, was seen as early as two weeks post-exchange transfusion. The whole group of newborns was followed up, at 3-monthly intervals for 12 months, with abdominal ultrasound and Doppler study and none of the remaining 126 cases developed EHPVO beyond the neonatal period. One of the 2 patients who developed PVT was diagnosed as Crigler-Najjar syndrome type 1 and underwent 3 exchange transfusions before starting home phototherapy. The second case also did exchange transfusion thrice for severe neonatal indirect hyperbilirubinemia and sepsis; her initial serum bilirubin on the second day of life was 35 mg/dl.^26^

Some hereditary causes for thrombophilia were studied at the Pediatric Hepatology Unit at Cairo University, Egypt, in a group of 40 children with EHPVO as possible risk factor for development of PVT (Table 1).^27^

They included factor V Leiden (FVL) mutation, Factor II (prothrombin G20210A) mutation, activated protein C resistance (APCR), assay of protein C, protein S and antithrombin and mutation in tetrahydrofolate reductase (MTFHR) gene (Table 2)^27^,^28^. Among the patients, 11 had protein C deficiency (27.5%), 1 was deficient in antithrombin (2.5%), none had protein S deficiency, APCR was detected in 12 cases (30%), the same 12 cases were heterozygous for FVL mutation, and none was homozygous.

Factor II mutation was found in six cases (15%); all were heterozygous. MTFHR C667T mutation was found in 4 cases (2 homozygous and 2 heterozygous). Only one of the controls was heterozygous for FVL mutation and had APCR, 4 were heterozygous for MTFHR C667T mutation and no other thrombophilia was detected in the control group. FVL mutation and APCR were significantly more prevalent in patients than controls (p=0.03), while no statistically significant difference was found between cases and controls as regards the prevalence of factor II mutation. The relative risk of development of PVT with FVL mutation was calculated using the odds ratio and was found to be 6, which is statistically significant (p<0.05). We concluded that hereditary thrombophilia was common in children with EHPVO (62.5%), the commonest being FVL mutation (30%). Concurrence of more than one hereditary thrombophilia is not uncommon (12.5%). Circumstantial risk factors were not more significantly prevalent among patients with hereditary thrombophilia than among those with no detectable abnormalities in anticoagulation.

## Hepatic Venous Outflow Obstruction:

Obstruction to the hepatic venous outflow includes sinusoidal obstruction syndrome also known as hepatic veno-occlusive disease (VOD) and venous obstruction from the level of the small hepatic veins to the junction of the inferior vena cava to the right atrium also known as Budd-Chiari syndrome (BCS).

The first report of hepatic VOD was in children. Four local reports from Egypt referred to children who rapidly develop abdominal distention with ascites and hepatomegaly. Dilated veins soon become apparent in their anterior abdominal walls, the spleen does not enlarge at the onset, and jaundice is either absent or slight.^29^,^30^,^31^,^32^

Professor Mohammad Safouh, the father of Pediatric Hepatology in Egypt, published his first international report on VOD in 1965.^33^ These cases were frequently seen at that time. They reported on 59 cases presenting to Cairo University Pediatric Hospital over 4 years. There ages ranged between 1–12 years, however, 47 patients were below 4 years; males/females were 36/23. All cases came from a poor social class and the majority lived in rural areas. Abdominal distention was the presenting feature. In 55 it was rapid in onset. The liver was large and firm as a rule. Ascites was always present and dilated veins were seen on the abdominal wall in the epigastrium and over the lower sternal border. The spleen usually was not enlarged. The weight and height were below average. Jaundice was absent or just a tinge. Lower limb edema was present in a third of the cases. Four cases were above 6 years of age and had a chronic form with marked enlargement of the liver and spleen, with little or no ascites. A history of preceding fever was reported shortly before the onset in half of the cases. Diarrhea, in the form of dysentery was not uncommon before the onset. Dietary survey done for 17 cases revealed reduced protein intake and excessive consumption of vegetables as well as plant seeds infusions. Changes in blood counts, erythrocyte sedimentation rate, serum proteins and transaminases were unremarkable, but the ascetic fluid was rich in protein. Sixteen autopsies were performed and revealed obliteration of the main hepatic veins and their ostia in the inferior vena cava by organized thrombi. Extension of the obliteration in the hepatic veins was variable. Liver showed advanced nutmeg appearance in acute cases. In one patient with advanced disease the liver was finely nodular. Liver histology showed centrilobular necrosis and fatty changes in the periphery of the lobules. Portal tract thickening was rarely marked. Management was in the form of high protein diet, tapping of ascites, which rapidly re-accumulated, diuretics and sometimes plasma transfusions. Some operative procedures were tried like omentopexy, hepatic artery ligation and laparotomy to relieve an obstructed hernia. Three chronic cases had a stationary course with disappearance of ascites. Most acute cases died within a few months. Hematemesis occurred in only one case. Jaundice rarely developed, and although jaundice often deepened before death, some patients died anicteric. This report concluded that this disease was more frequent in malnourished children coming from rural areas. Infusions given at home may contain noxious substances that were hepatotoxic. Infections might play a role. Senecio poisoning was reported with a picture of Chiari syndrome.^34^ However, the vegetables and drinks commonly ingested by Egyptian children were not of the Senecio class but might have contaminated them.

Classic hepatic VOD has become extremely rare in children since the 80s, when VOD became more commonly seen in adults as a complication of stem cell or bone marrow transplantation.^35^,^36^ This marked reduction in the incidence of the disease in Egyptian children may be attributed to improved sanitary and hygienic conditions that predispose to the disease.

## Conclusion:

In view of the deficient reporting on SVT from South Mediterranean countries, despite its deleterious effect on child health, both hereditary and acquired factors for thrombophilia and substantial risk factors need to be studied more in this region in this age group. Deficient data might also be attributable to reporting in local medical journals which are difficult to search for and access. Pediatric medical societies in countries of the region have to play a role in collecting available data and distributing information in a trial to reach a consensus in diagnosis and management of such rare but serious condition.

## Figures and Tables

**Table 1:** Characteristics of the 40 cases with EHPVO

**Age: mean (range)**	9.4 (1–15 years)
**Sex: ratio (M/F)**	2.1:1 (27/13)
**Main presenting symptoms**	
Hematemesis (n/%)	7/17.5%
Melena (n/%)	1/2.5%
Hematemesis and melena (n/%)	15/37.5%
Abdominal distention (n/%)	11/27.5%
Abdominal pain (n/%)	3/7.5%
Accidentally detected splenomegaly (n/%)	3/7.5%
**Circumstantial risk factors**	
Neonatal sepsis	2/10%
Umbilical sepsis	3/5%
Umbilical catheterization	2/5%
Severe gastroenteritis and dehydration	None
Family history of thromboembolism	None
**Consanguinity of parents**	13/32.5%
**Splenomegaly**	35/87.5%
**Splenectomy**	5/12.5%
**Ultrasound findings**	
Portal vein obstruction	33/82.5%
Portal vein cavernoma	7/17.5%
**Upper gastrointestinal endoscopy findings**	
Esophageal varices	40/100%
Eradicated varices by sclerotherapy	6/15%
Gastric varices	10/25%
Congestive gastropathy	3/7.5%

**Table 2:** Frequency of protein C, protein S, antithrombin deficiencies, APCR, and factor V and factor II mutations among the 40 cases with EHPVO.

**Thrombophilia**	**n**	**%**
**Single factor abnormality**	20	50
Protein C deficiency	8	20
Protein S deficiency	0	0
Antithrombin III deficiency	0	0
Factor V mutation + APCR	9	22.5
Factor II mutation	3	7.5
MTFHR C667T mutation	2	5
**Combined factors abnormalities**	5	12.5
Protein C deficiency + antithrombin III deficiency	1	2.5
Protein C deficiency + factor V mutation + APCR	1	2.5
Protein C deficiency + factor II mutation	1	2.5
Factor V mutation + APCR + factor II mutation	2	5
Factor V mutation + APCR + MTFHR C667T mutation	2	5
**Patients with no detectable abnormalities**	15	37.5
